# Steroid responsive encephalopathy in cerebral amyloid angiopathy: a case report and review of evidence for immunosuppressive treatment

**DOI:** 10.1186/1742-2094-7-18

**Published:** 2010-03-09

**Authors:** Raoul P Kloppenborg, Edo Richard, Marieke ES Sprengers, Dirk Troost, Piet Eikelenboom, Paul J Nederkoorn

**Affiliations:** 1 Department of Neurology, Academic Medical Center, Amsterdam, The Netherlands; 2 Department of Radiology, Academic Medical Center, Amsterdam, The Netherlands; 3 Department of Neuropathology, Academic Medical Center, Amsterdam, The Netherlands

## Abstract

Cerebral amyloid angiopathy (CAA) is a common but often asymptomatic disease, characterized by deposition of amyloid in cerebral blood vessels. We describe the successful treatment of CAA encephalopathy with dexamethasone in a patient with CAA-related inflammation causing subacute progressive encephalopathy and seizures, which is an increasingly recognized subtype of CAA. The two pathological subtypes of CAA-related inflammation are described and a review of the literature is performed concerning immunosuppressive treatment of CAA-related inflammation with special attention to its pathological subtypes. Immunosuppressive therapy appears to be an appropriate treatment for CAA encephalopathy.

## Background

Sporadic cerebral amyloid angiopathy (CAA) is a common but often asymptomatic neuropathological finding, characterized by the deposition of amyloid-β (Aβ) in small and medium-sized cerebral arteries, arterioles and sometimes capillaries of the meninges and brain parenchyma. Its prevalence is strongly associated with increasing age and has been reported to be as high as 57% percent in case series of asymptomatic patients over 60 years of age [1]. CAA is a common finding in patients with Alzheimer's disease (AD); but many patients with CAA do not develop AD. CAA can lead to lobar haemorrhage in non-hypertensive patients [2]. Other, less often reported clinical manifestations are seizures, transient neurological deficits and dementia other than AD [3]. In addition, more rare presentations have been reported, including space occupying lesions and leukoencephalopathy on magnetic resonance imaging (MRI) [4-6]. The latter is an increasingly recognized syndrome encompassing subacute encephalopathy, headache, seizures or focal neurological symptoms. Upon brain biopsy, an inflammatory process is found in relation to the vascular deposits of Aβ. In contrast to other Aβ-depositing disorders such as AD, immunosuppressive treatment has been reported to ameliorate both clinical and radiological symptoms of CAA encephalopathy, although with variable success [7]. This variability could be explained by the existence of different underlying pathological subtypes. We describe a patient with CAA-leukoencephalopathy, who was treated successfully with dexamethasone. We also performed a literature review concerning the use of immunosuppressive treatment for CAA-related inflammation with special attention to its pathological subtypes.

## Case presentation

**Figure 1:**
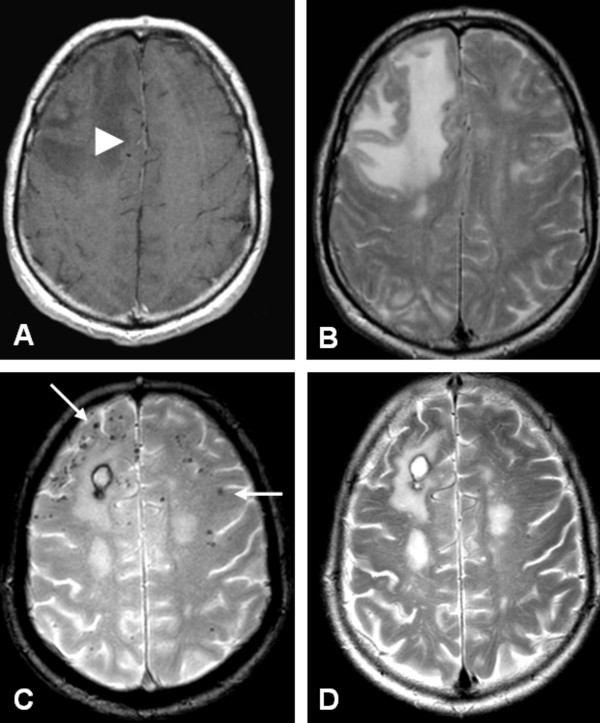
**Axial MRI at presentation (A, B) and 3 months after treatment (C, D)**. A) Contrast enhanced, T1- weighted image shows low signal intensity of the right frontal lobe with minimal enhancement of the white matter (white arrowhead). B) T2-weighted image shows high signal intensity in the right frontal lobe. C) Gradient echo sequence shows subcortical 'black dots', consistent with microbleeds (white arrows), and a small postoperative hematoma after biopsy. D) T2-weighted image after treatment shows a decrease of high signal intensity in the right frontal lobe.

**Figure 2:**
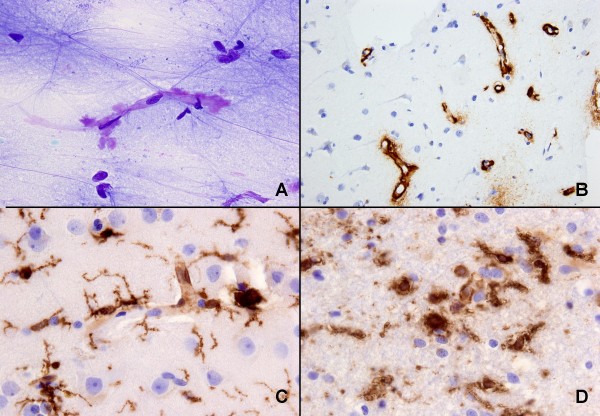
**Cytological and histological examination of biopsy**. A) Smear slide showing amyloid (metachromatic, purple) around a capillary (toluidin blue stain), B) paraffin-embedded material: extensive amyloid deposition around capillaries in cortex (Aβ immunoreaction), C) reactive gliosis and upregulation of microglia and macrophages in grey matter, D) reactive gliosis, upregulation of microglia and presence of macrophages in white matter (C and D, HLA-DR (CR3/43) immunoreaction).

A 74-year-old man with an unremarkable medical history noted a progressive gait disorder in the months prior to admission. His wife recalled increased sleepiness and loss of initiative. After having seizures the patient was admitted to our hospital. The patient was disorientated in time and did not perform complicated tasks, although this could partly be attributed to apathy. He could not remember the reason for his stay in the hospital. The remaining neurological examination revealed no abnormalities. MRI showed confluent bifrontal white matter lesions and minimal enhancement of the white matter in the right frontal lobe after administration of gadolinium (Figure 1A-D). Routine laboratory measurements were normal. Cerebral spinal fluid examination revealed an elevated protein level (1.78 g/l). No malignant cells were found in the spinal fluid. After diagnostic work-up had excluded a primary tumour elsewhere in the body, low grade astrocytoma or gliomatosis cerebri was considered and a stereotactic brain biopsy was performed. Histopathological analysis showed extensive Aβ immunopositivity around smaller and larger blood vessels (Figure 2A, B). No neurofibrillary tangles or amyloid plaques were found in the parenchyma. Reactive gliosis, strong upregulation of microglia and multiple macrophages around the blood vessels in both white and grey matter were present (Figure 2C, D). The findings were compatible with sporadic CAA. After the patient developed progressive apathy, loss of initiative, magnetic gait and hypertonia of the extremities, treatment with dexamethasone (2 × 4 mg/day) was started. There was a remarkable clinical improvement in the following days. The patient became alert, the hypertonia disappeared and he was able to walk with a wheeled walker. After 5 weeks, he was discharged from the hospital with a mild gait disorder. A 3 Tesla MRI three months after admission showed remarkable amelioration of the white matter abnormalities. Gradient echo sequences showed subcortical hypointensities, compatible with multiple microbleeds (figure 1). Dexamethasone treatment was tapered in the months after admission.

## Discussion

The clinical picture of CAA-related inflammation includes encephalopathy, seizures and headaches. Extensive vasogenic edema and/or leukoencephalopathy is visible on MRI, sometimes mimicking space-occupying lesions. Histological examination shows amyloid-laden vessels and the appearance of Aβ in close association with inflammatory cells, implicating Aβ as the potential trigger for the inflammatory response. It remains unclear why only a few CAA patients develop this response. A high percentage of such patients are homozygous for the ε4-allele of the apolipoprotein E gene (APOE ε4/ε4; 76.9% vs 5.1% in non-inflammatory CAA) [6], which is associated with activation of complement and microglia. Additionally, trials of anti-Aβ vaccination in patients with Alzheimer's disease (AD) induced similar clinical, radiological and pathological inflammation as seen in CAA-related inflammation, suggesting an immune response to Aβ.

Unlike other Aβ-depositing disorders, CAA-associated inflammation appears to derive a beneficial effect from corticosteroid treatment. This effect could be dependant on the pathological subtype of CAA-related inflammation.

Two subtypes of CAA-associated inflammation have been described so far: (i) a non-vasculitic form called perivascular infiltration (PVI), which is characterized by perivascular infiltration of the parenchyma by multinucleated giant cells and (ii) a vasculitic form called transmural (non)-granulomatous angiitis (TGA), which is characterized by inflammation of the vessel wall with the occasional presence of granulomas. Both pathologic forms can co-occur, suggesting at least a partial overlap [8]. The clinical and radiological findings of both variants are remarkably similar. Our case showed reactive gliosis and multiple macrophages around blood vessels in grey and white matter, although no multinucleated cells were seen. This is consistent with reactive edema in encephalopathy and suggests PVI. Although often called CAA-angiitis, the terms CAA-vasculopathy or CAA-encephalopathy are preferred, since these terms do not exclude the considerable numbers of cases with only perivascular inflammation [8].

Because of various reports regarding success of corticosteroid treatment, we performed a literature review on the use of immunosuppressive agents in CAA-encephalopathy with special attention to its pathological subtypes. A total of 45 patients in 18 articles could be identified (Table 1) [4-6,8-22]. In four patients TGI and PVI co-occurred [8,15,18]. Corticosteroids were the most commonly used drugs, varying from short, high-dosage intravenous treatments to continuous treatment with low-dose prednisone. One case report reported effective treatment with low dose cyclophosmamid alone [16]. Similar to our case, there were generally favourable outcomes in most patients after immunosuppressive treatment. Clinical and radiological symptoms were (partly) reversed in 76% of all patients, although some experienced relapses during follow-up.

**Table 1:** Studies concerning immunosuppresive treatment of CAA encephalopathy

Author	n	Age	Pathology	Radiology	Therapy	Clinical improvement	Radiological improvement	Follow-up	Clinical features	Comments
Ginsberg 1988 [10]	1	73	TGA	Confluent	Dx, Pn	Yes	Yes	>1 year	Gait disturbance	

Mandybur 1992 [11]	1	62	TGA	Mass	CP, Pn	Yes	Yes	Death 8 months	Encephalopathy Focal neurology Hallucinations	Remarkable pathological improvement lesions post-mortem compared to initial biopsy

Osumi 1995 [4]	1	59	?	Mass	CS	No	?	Death 5 months	Focal neurology Headaches Seizures	

Silbert 1995 [12]	1	74	?	Confluent	Dx	No	No	Death 6 weeks	Headache Seizures	

Fountain 1996 [13]	1	66	TGA	Confluent	CP, Dx, Pn,	No	Partial	20 months	Encephalopathy Headaches Seizures	

Fountain 1996 [13]	1	69	TGA	Confluent	CP, Dx, Pn,	Partial	Partial	Death 6 months	Encephalopathy Headaches Seizures	Relapse

Ortiz 1996 [14]	1	68	PVI	Mass	Dx, Pn	Yes	Yes	?	Encephalopathy Gait disturbance Headaches	

Masson 1998 [15]	1	64	PVI	Confluent	CP, Pn	Yes	No	15 months	Encephalopathy Headaches	

Fountain 1999 [16]	1	71	PVI, TGA	Confluent	CP	Yes	Yes	22 months	Encephalopathy Gait disturbance Headaches	Relapse after stop CP

Streichenberger 1999 [17]	1	67	TGA	Mass/Confluent	CS	Yes	Yes	Death 1 month	Headaches Encephalopathy	

Hoshi 2000 [18]	1	65	- (after treatment)	Recurrent ICH	Pn	Yes	NA	6 months	Focal neurology	

Schwab 2003 [19]	1	74	PVI/TGA	Mass	Dx 1m, Pn	Yes	?	12 months	Encephalopathy Headaches Seizures	

Schwab 2003 [19]	1	70	PVI//TGA	Mass	Pn, CP	Partial	Yes	18 months	Encephalopathy Headaches Seizures	

Oh 2004 [20]	1	80	PVI	Confluent	Dx, Pn	Yes	Yes	8 months	Encephalopathy Focal Neurology Seizures	1 patient with no therapy excluded

Oh 2004 [20]	1	77	TGA	Confluent	Dx, Pn	Yes	Yes	6 weeks	Encephalopathy Focal Neurology Seizures	1 patient with no therapy excluded

Safriel 2004 [5]	1	49	TGA	Mass	Dx, tap 6 weeks	Yes	Partial	9 months	Seizures	

Scolding 2005 [21]	7	69*	TGA	Confluent	Pn, Dx, CP	43%	?	58 months*	Encephalopathy Focal Neurology Headaches Seizures	2 patients excluded because of mass resection as therapy

Kinnecom 2007 [6]	12	63.2 ± 10	PVI	Confluent	CS, CP	83%	83%	47 months*	Encephalopathy Headache Seizures	25% relapse, 33% died

McHugh 2007 [9]	1	80	PVI, TGA	Confluent	Pn	Yes	Yes	24 months	Encephalopathy Focal Neurology Seizures	

Machida 2008 [22]	1	69	PVI	Confluent	Dx, Pn	Yes	Yes	12 months	Encephalopathy Focal neurology	Relapsing/remitting

Salvarini 2008 [23]	8	63	TGA	Confluent	Pn, CP	75%	100%	24 months*	Encephalopathy Focal Neurology Headaches	25% relapse, both after discontinuation of treatment

CS = corticosteroid (not otherwise specified), CP = Cyclophospamid, Dx = dexamethasone, Pn = prednisone, PVI = perivascular inflammation, TGA = transmural (non)-granulomatous angiitis

Encephalopathy is characterised as diffuse cognitive disturbances, somnolence and apathy, focal neurology is characterised as hemiparesis, hemihyesthesia, aphasia or hemianopsy.

* calculated mean

Although publication bias, small study numbers and the possible self-limiting nature of the disease has to be taken into account, the quick response to immunosuppressive therapy and the tendency to relapse in drug-free periods suggest a beneficial effect of immunosuppressants. In general, patients with TGA did not benefit as much as patients with PVI [68% vs 88%]: possibly the vasculitic form gives rise to more ischemic lesions (reflected in the higher proportion of focal deficits in this group) than in PVI, in which there could be more vasogenic edema.

It is interesting that only CAA encephalopathy responds to corticosteroid treatment, in contrast to other Aβ-depositing disorders. A possible explanation could be that corticosteroids merely reduce cerebral vasogenic edema. However, in vitro research has shown that dexamethasone diminishes the pro-inflammatory and cytotoxic effects of Aβ in cerebrovascular smooth muscle cells in later stages of the inflammation process, although it does not affect initial Aβ deposition [23]. This suggests a direct effect upon the pathogenesis of acute inflammation in Aβ-disorders, as it is found in CAA encephalopathy. In any case, our review shows there is a possible role for the use of corticosteroids in patients with CAA encephalopathy.

## Conclusions

CAA encephalopathy is an increasingly recognized syndrome that is based upon a vasculitic or non-vasculitic inflammatory reaction to Aβ. Although the clinical and radiological symptoms are similar in both pathologic variants, immunosuppressive therapy appears to have a slightly less beneficial effect in the vasculitic subtype. Nevertheless, corticosteroid therapy seems to be an appropriate therapy for both. In an elderly patient with a subacute progressive encephalopathy with seizures, CAA-related encephalopathy has to be considered because of the major therapeutical implications.

## Consent

Written informed consent was obtained from the next of kin of the patient for publication of this case report and accompanying images. A copy of the written consent is available for review by the Editor-in-Chief of this journal.

## Competing interests

The authors declare that they have no competing interests.

## Authors' contributions

RPK participated in the design of the article, collected and analyzed the data and drafted the manuscript. ER, PE and PJN contributed to the analysis and interpretation of the data. MES and DT provided radiological and pathological data respectively. PJN conceived of the case report and coordinated the drafting of the manuscript. All authors read and approved the final manuscript.
